# Efficient Syntheses of [(*n*-C_4_H_9_)_4_N]_4_[α-Mo_8_O_26_] and [(*n*-C_4_H_9_)_4_N]_2_[Mo_2_O_7_]

**DOI:** 10.3390/ma2030869

**Published:** 2009-07-28

**Authors:** Shusaku Ikegami, Atsushi Yagasaki

**Affiliations:** Department of Chemistry, Kwansei Gakuin University, Sanda 669-1337, Japan

**Keywords:** α-octamolybdate, dimolybdate, synthesis, tetra-*n*-butylammonium

## Abstract

Efficient and simple syntheses of [(*n*-C_4_H_9_)_4_N]_4_[α-Mo_8_O_26_] (**I**) and [(*n*-C_4_H_9_)_4_N]_2_[Mo_2_O_7_] (**II**) from MoO_3_ and aqueous [(*n*-C_4_H_9_)_4_N]OH are described. The yield is 72% for **I** and 73% for **II**.

## 1. Introduction

The tetrabutylammonium salts of [α-Mo_8_O_26_]^4-^ [1,2,3] and [Mo_2_O_7_]^2-^ [4] are important starting materials in the synthesis of variety of polyoxometalates and metal-organic hybrid materials. By virtue of their solubility in aprotic organic solvents, these salts have found widespread application [5,6,7,8,9,10,11,12,13,14,15,16,17,18,19,20,21,22,23,24,25,26,27]. In the method currently employed for the preparation of [(*n*-C_4_H_9_)_4_N]_4_[α-Mo_8_O_26_] (**I**) (Scheme 1), Na_2_MoO_4_ and [(*n*-C_4_H_9_)_4_N]Br are used as starting materials [28]. Because of this, large amounts of sodium salts are produced as byproducts.

**Scheme 1:** Classical synthesis of [(*n*-C_4_H_9_)_4_N]_4_[α-Mo_8_O_26_] (**I**).

8Na_2_MoO_4_ + 12HCl + 4[(*n*-C_4_H_9_)_4_N]Br → [(*n*-C_4_H_9_)_4_N]_4_[α-Mo_8_O_26_] + 12NaCl + 4NaBr + 6H_2_O

These byproducts tend to make the final yield low and to fluctuate from one preparation to another. In order to prepare [(*n*-C_4_H_9_)_4_N]_2_[Mo_2_O_7_] (**II**), one has to first prepare **I** (Scheme 2) [28].

**Scheme 2:** Classical synthesis of [(*n*-C_4_H_9_)_4_N]_2_[Mo_2_O_7_] (**II**).

[(*n*-C_4_H_9_)_4_N]_4_[α-Mo_8_O_26_] + 4[(*n*-C_4_H_9_)_4_N]OH → 4[(*n*-C_4_H_9_)_4_N]_2_[Mo_2_O_7_] + 2H_2_O

Although the conversion rate is moderate (69%), the need to first prepare **I** makes the overall yield of **II** based on the initial starting material, Na_2_MoO_4_, as low as 44%. We report here preparations that are more convenient and more efficient than those currently employed.

## 2. Results and Discussion

Compound **I** was efficiently synthesized by mixing MoO_3_ and [(*n*-C_4_H_9_)_4_N]OH (Scheme 3).

**Scheme 3:** New synthesis of [(*n*-C_4_H_9_)_4_N]_4_[α-Mo_8_O_26_] (**I**).

8MoO_3_ + 4[(*n*-C_4_H_9_)_4_N]OH → [(*n*-C_4_H_9_)_4_N]_4_[α-Mo_8_O_26_] + 2H_2_O

The pH of the suspension decreased from 7.8 to 6.1 as the reaction proceeded and the solid acid consumed OH^-^. The color of the suspension also changed during this period from light gray to bright white. Compound **I** is insoluble in water, and it seems that what little amount of **I** formed by the reaction of MoO_3_ and [(*n*-C_4_H_9_)_4_N]OH immediately precipitated out from the solution. In fact, the powder obtained by filtering the suspension after its color changed to bright white gave IR spectra identical to that of pure **I**. When the molar ratio of [(*n*-C_4_H_9_)_4_N]OH to MoO_3_ was increased to 1, **II** was obtained in high yield (Scheme 4).

**Scheme 4:** New synthesis of [(*n*-C_4_H_9_)_4_N]_2_[Mo_2_O_7_] (**II**).

2MoO_3_ + 2[(*n*-C_4_H_9_)_4_N]OH → [(*n*-C_4_H_9_)_4_N]_2_[Mo_2_O_7_] + H_2_O

Again, the pH of the suspension decreased from 7.5 to 6.6 and the color changed to white as the reaction proceeded. However, the solid left undissolved in this aqueous suspension was not **II** and the IR spectrum showed that it was actually **I**. This observation suggests that **II** does not form when the reaction medium is water, even if the molar ratio of [(*n*-C_4_H_9_)_4_N]OH to MoO_3_ is optimal for its formation. It seems that, even in such a case, **I** forms first and it then reacts with the remaining [(*n*-C_4_H_9_)_4_N]OH to form [(*n*-C_4_H_9_)_4_N]_2_[MoO_4_], which is highly soluble in water. In other words, the substance obtained by evaporating the suspension was probably a mixture of **I** and [(*n*-C_4_H_9_)_4_N]_2_[MoO_4_]. Compound **II** was obtained only after re-dissolving the substance in acetonitrile, whereupon **I** reacts with [(*n*-C_4_H_9_)_4_N]_2_[MoO_4_] to form **II** (Scheme 5)

**Scheme 5:** Formation of [(*n*-C_4_H_9_)_4_N]_2_[Mo_2_O_7_] (**II**).

4[(*n*-C_4_H_9_)_4_N]_2_[MoO_4_] + [(*n*-C_4_H_9_)_4_N]_4_[α-Mo_8_O_26_] → 6[(*n*-C_4_H_9_)_4_N]_2_[Mo_2_O_7_]

**Figure 1:**
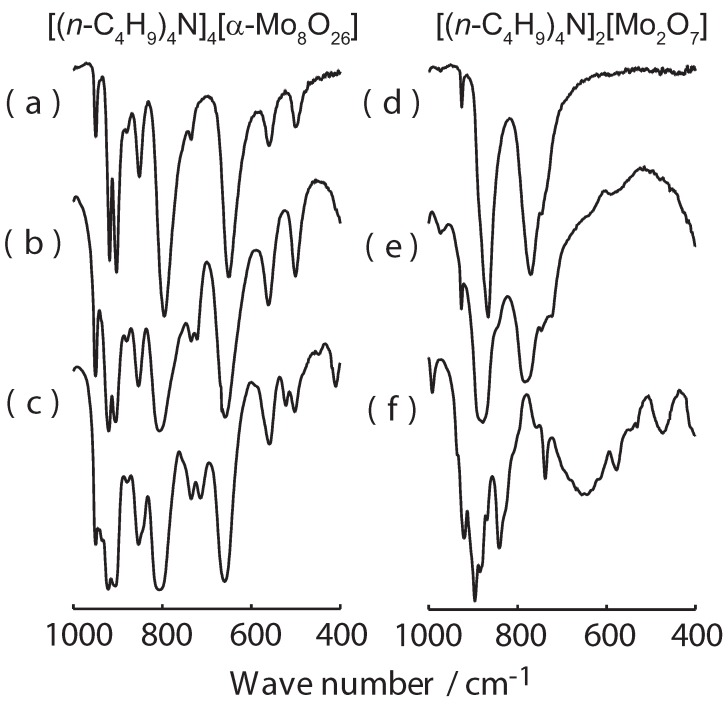
IR spectra of [(*n*-C_4_H_9_)_4_N]_4_[α-Mo_8_O_26_] (**I**, left column) and [(*n*-C_4_H_9_)_4_N]_2_[Mo_2_O_7_] (**II**, right column) from ATR [(a) and (d)], Nujol mull [(b) and (e)], and KBr pellet [(c) and (f)].

Both compounds **I** and **II** prepared by the current method gave satisfactory elemental analyses. They both have characteristic IR absorptions in the 1,000–400 cm^-1^ range and thus can be conveniently identified by their IR spectra (Figure 1). However, care should be taken when measuring these IR spectra, as compounds **I** and **II** both react with KBr under pressure and give different spectra when they are recorded as KBr pellets [29].

The current reactions for preparing **I** and **II** proceed relatively slowly because they are heterogeneous reactions. However, the overall time necessary to obtain analytically pure **I** in a crystalline form is not very different from that of the conventional method. The time required to obtain **II** is much shorter and the overall yield has been improved significantly in the current method, because there is no need to prepare **I** first.

In addition to providing simple and efficient methods for synthesizing **I** and **II**, the current study demonstrates that MoO_3_ is a good starting material for obtaining polymolybdates soluble in aprotic organic solvents, although MoO_3_ itself is insoluble in those solvents. In fact, [(*n*-C_4_H_9_)_4_N]_5_[IMo_6_O_24_] has recently been prepared in a good yield using MoO_3_ as a starting material [30].

## 3. Experimental

### 3.1. General

The following were purchased from commercial sources and used without further purification: MoO_3_ (Nakarai), P_2_O_5_ (Kishida), and 10% aqueous [(*n*-C_4_H_9_)_4_N]OH (Wako). Acetonitrile (Wako) was dried over 3-Å molecular sieves. Diethyl ether (Kishida) was dried over 4-Å molecular sieves. The pH of the reaction mixtures was measured with METTLER DELTA340 pH meter. IR spectra were recorded on a Shimadzu FTIR-8400 spectrometer. The numerical values given below are for the spectra recorded from mineral oil (Nujol) mulls between KBr plates. Absorptions are described as follows: very strong (vs), strong (s), medium (m), weak (w), and shoulder (sh). Elemental analyses were performed by Toray Research Center Inc., Shiga, Japan.

*Preparation of [(n-C_4_H_9_)_4_N]_4_[α-Mo_8_O_26_]* (**I**): Molybdenum(VI) oxide (MoO_3_, 1.2 g, 8.3 mmol) was mixed with aqueous [(*n*-C_4_H_9_)_4_N]OH (10 mL, 4.1 mmol). The mixture was stirred for 60 h, during which time the pH of the mixture went down from 7.8 to 6.1 and the slightly gray suspension turned bright white. The precipitate was collected on a fine-porosity filter with suction and dried *in vacuo* over P_2_O_5_. This crude product (2.1 g) was dissolved in 15 mL of acetonitrile. After a small amount of insoluble material was filtered off with a fine-porosity filter, the filtrate was stored overnight at – 35 °C. The clear, colorless, block-shaped crystals that formed were collected on a medium-porosity filter with suction and dried *in vacuo* over P_2_O_5_ for 8 h to yield 1.6 g of the product (0.74 mmol, 72%). Anal. calcd. for C_64_H_144_N_4_Mo_8_O_26_: C, 35.70; H, 6.74 N, 2.60; Mo, 35.6. Found: C, 35.67, H, 6.75; N, 2.77; Mo, 35.6. IR (400 – 1,000 cm^-1^): 501 (m), 562 (m), 659 (vs), 722 (m), 735 (m), 807 (s), 854 (m), 881 (w), 905 (s), 920 (s), 950 (m).

*Preparation of [(n-C_4_H_9_)_4_N]_2_[Mo_2_O_7_]* (**II**): Molybdenum(VI) oxide (MoO_3_, 0.59 g, 4.1 mmol) was mixed with aqueous [(*n*-C_4_H_9_)_4_N]OH (10 mL, 4.1 mmol). The mixture was left stirring overnight, during which time the pH of the mixture went down from 7.5 to 6.6 and the slightly gray suspension turned bright white. The mixture was then evaporated to dryness under vacuum, and the solids thus obtained were dissolved in acetonitrile (5 mL). After a small amount of insoluble material was filtered off with a fine porosity filter, diethyl ether (23 mL) was added to the filtrate. The mixture was then stored overnight at –35 °C. The colorless crystals that formed were collected on a medium-porosity filter with suction and dried *in vacuo* over P_2_O_5_ for 4 h to yield 1.2 g of the product (1.5 mmol, 73%). Anal. calcd. for C_32_H_72_N_2_Mo_2_O_7_: C, 48.73; H, 9.20 N, 3.55. Found: C, 48.63, H, 9.33; N, 3.64. IR (400–1,000 cm^-1^): 746 (m), 770 (s), 867 (vs), 927 (w).

## 4. Conclusions

Direct reactions between MoO_3_ and [(*n*-C_4_H_9_)_4_N]OH in stoichiometric ratios have been proven to be a facile and convenient way to prepare tetra-*n*-butylammonium salts of [α-Mo_8_O_26_]^4-^ and [Mo_2_O_7_]^2-^ in high yields. The current study, together with the report of the successful synthesis of the tetra-*n*-butylammonium salt of [IMo_6_O_24_]^5-^ by a similar method [30], demonstrates the potential of MoO_3_ as a starting material for the general syntheses of non-aqueous polymolybdates.
